# Highly efficient transduction of human plasmacytoid dendritic cells without phenotypic and functional maturation

**DOI:** 10.1186/1479-5876-7-10

**Published:** 2009-01-27

**Authors:** Philippe Veron, Sylvie Boutin, Samia Martin, Laurence Chaperot, Joel Plumas, Jean Davoust, Carole Masurier

**Affiliations:** 1 Laboratoire d'Immunologie, GENETHON, CNRS UMR 8115, 91002 EVRY Cedex, France; 2 GENOSAFE SA, 91002 EVRY Cedex, France; 3 Service EFS Rhône-Alpes, La Tronche, F-38701 Inserm, U823, Immunobiologie et Immunothérapie des cancers, La Tronche, F-38706, Univ Joseph Fourier, Grenoble, F-38041 France; 4 INSERM U580, Hôpital Necker-Enfants-Malades, Université Paris Descartes, Faculté de Médecine René Descartes, 75015 Paris, France

## Abstract

**Background:**

Gene modified dendritic cells (DC) are able to modulate DC functions and induce therapeutic immunity or tolerance in an antigen-specific manner. Among the different DC subsets, plasmacytoid DC (pDC) are well known for their ability to recognize and respond to a variety of viruses by secreting high levels of type I interferon.

**Methods:**

We analyzed here, the transduction efficiency of a pDC cell line, GEN2.2, and of pDC derived from CD34+ progenitors, using lentiviral vectors (LV) pseudotyped with different envelope glycoproteins such as the vesicular stomatitis virus envelope (VSVG), the gibbon ape leukaemia virus envelope (GaLV) or the feline endogenous virus envelope (RD114). At the same time, we evaluated transgene expression (E-GFP reporter gene) under the control of different promoters.

**Results:**

We found that efficient gene transfer into pDC can be achieved with VSVG-pseudotyped lentiviral vectors (LV) under the control of phoshoglycerate kinase (PGK) and elongation factor-1 (EF1α) promoters (28% to 90% of E-GFP^+ ^cells, respectively) in the absence of phenotypic and functional maturation. Surprisingly, promoters (desmin or synthetic C5–12) described as muscle-specific and which drive gene expression in single strand AAV vectors in gene therapy protocols were very highly active in pDC using VSVG-LV.

**Conclusion:**

Taken together, our results indicate that LV vectors can serve to design pDC-based vaccines in humans, and they are also useful *in vitro *to evaluate the immunogenicity of the vector preparations, and the specificity and safety of given promoters used in gene therapy protocols.

## Background

Dendritic cells (DC) are antigen-presenting cells (APC) with a role in controlling the balance between immunity and immunological tolerance [1,2]. In humans, at least two subsets of DC are known in the blood, myeloid DC (also known as interstitial or dermal DC), and plasmacytoid DC (pDC) and Langerhans cells (LC) in the tissues [3]. Plasmacytoid DC also called "natural interferon producing cells" (NIPC), represent 0.2–0.8% of peripheral blood cells and have also been found in the spleen, bone marrow, tonsils, lymph nodes, foetal liver and thymus [2,4-6]. Plasmacytoid DC are well known for their ability to recognize and respond to a variety of viruses [6]. They recognize viral genomic nucleic acids of dsDNA viruses [7-10] and ssRNA viruses [11-13] via Toll-like receptor 9 (TLR9) and TLR7, respectively in the acidified endosomes without becoming infected themselves. Plasmacytoid DC are characterized by their high secretion levels of type I interferon in response to viruses [14,15], which not only have direct inhibitory effects on viral replication, but also can promote the function of natural killer cells, B cells, T cells and myeloid DC [16]. Human pDC do not express lineage specific markers, but are characterized by the expression of HLA-DR, CD4, CD123, BDCA2 and BDCA4 [3]. These scarce cells can be generated from CD34^+ ^progenitor cells [17]. Recently, a pDC cell line called GEN2.2 established from leukemic pDC was described as sharing most of the phenotypic and functional features of normal pDC [18] and so represents a good model for study of the physiology of their normal counterpart [19].

Over the classical antigen loading methods usually considered, such as peptide or protein loading, gene modified DC offer potential advantages: 1) they ensure long-lasting expression of the antigen and production of an entire array of epitopes presented by the autologous HLA molecules, 2) antigens are delivered to both endogenous MHC class I and class II antigen presentation pathways [2,20]. Lentiviral vectors (LV) pseudotyped with the vesicular stomatitis virus envelope glycoprotein (VSVG) are efficient gene delivery vectors for dividing and non-dividing cells and were shown to be applicable to many cell types including human conventional DC and LC [21-26]. Transduced DC and LC retained their immature phenotype, were able to respond to maturation signals, and maintain immunostimulatory potential in both autologous and allogeneic settings [22,26,27]. To our knowledge, the transduction capacity of LV into pDC has not yet been evaluated. LV can be pseudotyped with a variety of envelope glycoproteins [28,29] such as the gibbon ape leukaemia virus envelope (GaLV) or the feline endogenous virus envelope (RD114) which have been reported to be efficient in the transduction of hematopoietic cells [30-32]. The elongation factor-1α (EF1α) and phoshoglycerate kinase (PGK) promoters were shown to have an activity in a human CD34^+ ^cell and in cultured cord blood cells and transgene-expressing myeloid DC were obtained from them [23,26,33,34].

One of the alternate vectors used to transduce monocytes or DC was the recombinant adeno-associated virus (rAAV) with a genome conventionally packaged as single-stranded molecules (ss) [35-37], characterized by its ability to transduce both dividing and non-dividing cells. Recombinant AAV is unique among viral vectors that are being developed for gene therapy applications in that the wild-type virus counterpart has never been shown to cause human disease. So far, transduction efficiencies of DC subsets have been shown to be low and variable [36,38].

In this study, we compared the transduction efficiency into a human pDC cell line and in CD34-pDC, with i) LV pseudotyped with different envelopes encoding E-GFP. In this context, we also tested different promoters: two promoters with high activity in hematopoietic cells (PGK and EF1a) and two promoters described as muscle-specific [39-41] (C5–12 and desmin) in order to evaluate the promoter leak in pDC, ii) rAAV of different serotypes. We found that efficient gene transfer into pDC can be achieved mainly with VSVG-pseudotyped LV under the control of PGK and EF1 promoters. Surprisingly, promoters described as muscle-specific were also highly active in pDC. Gene transfer into pDC could be of high importance for the design of new DC-based vaccines, or for induction of peripheral tolerance for dedicated therapeutic applications.

## Methods

### Culture of pDC line

Gen2.2 is a pDC cell line derived from a leukaemia patient. Tumor cells were characterized as pDC like [18]. Briefly, they grow on a murine fibroblast feeder cell line MS5 in RPMI, 10% FCS complemented with 1% L-glutamine, non essential amino acids, gentamycin and 0.2% sodium pyruvate.

### Lentiviral vector constructions and production

The VSV-G pseudotyped LV vectors were produced in 293 T cells by transient transfection of three plasmids, the transfer vector (pRRL-SIN-PPT-hPGK-GFP-WPRE, pRRL-SIN-PPT-hEF1-GFP-WPRE, pRRL-SIN-PPT-C512-GFP-WPRE, or pRRL-SIN-PPT-desmin-GFP-WPRE or pRRL-SIN-PPT-C512-MART1-WPRE the packaging construct pCMVΔR8.74 and the vesicular stomatitis virus envelope-expressing construct pMD.G. High-titer stocks were prepared by ultracentrifugation as described [42]. Also, GALV-pseudotyped LV vectors or RD114-pseudotyped LV vectors were produced in 293 T cells by transient transfection of the transfer vector pRRL-SIN-PPT-hPGK-GFP-WPRE, the packaging construct pCMVΔR8.74 and either the gibbon ape leukaemia virus chimeric envelope plasmid (pBA-GaLV-ampho) or feline leukaemia virus type C chimeric envelope plasmid (pBA-RD114-ampho). Vector supernatants were also concentrated by ultracentrifugation. Expression titers were determined by flow cytometry (FACSCalibur, Becton Dickinson, Mountain View, CA), on C2C12 cells for LV constructs with desmin and C5–12 promotors, and on HCT116 cells for the other constructs. Titers were 7.7 × 10^7 ^to 7.9 × 10^9 ^transducing units/ml.

### AAV vector construction and production

Pseudotyped AAV vectors were generated by packaging AAV2-based recombinant genomes in AAV1, AAV2 or AAV5 capsids. All the vectors used in the study were produced using the three-plasmid transfection protocol as described elsewhere [43]. Briefly, HEK293 cells were tri-transfected with the adenovirus helper plasmid pXX6 [44], a pAAV packaging plasmid expressing the *rep *and *cap *genes (pACG2.1 for AAV2, pLT-RC02 for AAV1 and pLT-RC03 for AAV5) and the relevant pAAV2 vector plasmid. ssAAV vectors were produced with conventional pGG2 AAV2 vector plasmid expressing E-GFP under the transcriptional control of the cytomegalovirus immediate early (CMV IE) promoter associated with a SV40 polyA signal. Recombinant vectors were purified by double-CsCl_2 _ultracentrifugation followed by dialysis against sterile phosphate-buffered saline (PBS). Physical particles were quantified by real time PCR and vector titers are expressed as viral genomes per ml (vg/ml).

### Cell line

The OP9 stroma cell line coding for human delta 1 (OP9-Del1) was kindly provided by A. Galy (Genethon, Evry, France) and maintained as previously described [17].

### Culture of peripheral blood monocytes and CD34^+ ^progenitors

Monocytes were generated from normal volonteers' monocytes after elutriation of peripheral blood according to the french EFS procedures (Pr Jacky Bernard, Reims, France). This method yielded purified (92.2% +/- 5.1) CD14^+^CD45^+ ^cells as assessed by flow cytometry. Briefly, cryopreserved monocytes were cultured in 6-well plates, at a density of 1 × 10^6 ^cells/ml in RPMI 1640 (Invitrogen Life technology, Auckland, USA) supplemented with 10% of FCS (Hyclone, Logan, Utah, USA) and 1% L-glutamin (Invitrogen). Monocytes were differentiated in cDC (Mo-DC) in presence of 50 ng/ml of recombinant human (rh) GM-CSF (Novartis, Bâle, Switzerland), and 15 ng/ml of rhIL-4 (Tebu-bio, le Perray, France). Maturation was induced in some experiments by addition of LPS (7 μg/ml Sigma-aldrich, St.Louis, MO, USA) at day 8, for 24 hours.

pDC were generated from cord blood CD34^+ ^cells (CD34-pDC) following protocols previously described by Olivier et al [17]. 2 × 10^4 ^CD34^+ ^progenitors were added onto OP9-Del1 cells seeded one day before, in 24-well plates at 3 × 10^4 ^cells/well. Cells were cultured in RPMI 1640 (Invitrogen) supplemented with 10% FCS (Hyclone), 1% L-glutamine and 1% Penicillin/Streptomycin (Gibco) in the presence of recombinant human Fms-like tyrosine kinase-3-Ligand (FLT3-L; 5 ng/ml) and rIL-7 (5 ng/ml; R&D Systems, Minneapolis, MN). Maturation of CD34-pDC was induced in some experiments by addition of CpG oligodeoxynucleotide type A (ODN 2216 at 2 μM) at day 10, for 24 hours. All cells were cultured in a humidified incubator at 37°C and 5% CO_2_.

### Transduction of GEN2.2

GEN2.2 were transduced by lentiviral vectors at multiplicity of infection (MOI) of 18 TU/ml or adeno-associated vector at 9 × 10^3 ^to 25 × 10^3 ^viral genome (Vg)/cell. Transductions were carried out just after thawing at a fixed concentration of 2–5 × 10^6 ^of cells per 200–500 μl of medium. After 3 hours at 37°C, cells were placed in complete medium and analysed by flow cytometry between day 5 and day 60.

### Transduction of CD34-pDC

Semi-adherent and non-adherent cells in culture were harvested at day 6 and transduced by LV-VSVG-PGK at an MOI of 18 TU/ml and at a fixed concentration of 1 × 10^6 ^cells/ml, in RPMI 1640. After 3 hours at 37°C, cells were replaced in the same complete medium then cultured for 5–6 additional days.

### Transduction of monocytes

After thawing, monocytes were transduced by LV-VSVG-PGK at an MOI of 18 TU/ml and at a fixed concentration of 1 × 10^6 ^cells/ml respectively, in RPMI 1640. After 3 hours at 37°C, cells were cultured in complete medium as described above to generate Mo-DC.

### ELISA

Human interferon-α levels were determined using specific ELISA kit (R&D Systems, Minneapolis, MN). Lower limit of detection was 10 pg/ml.

### Mixed leukocyte reaction (MLR)

Enriched naïve CD45RA+ T-cells were recovered after elutriation of monocytes. This method yielded purified (83.6% +/- 7.3) CD45RA+cells as assessed by flow cytometry. CD45RA+T cells were labelled with carboxyfluorescein diacetate succinimidyl ester (CFSE) at a final concentration of 0.5 μM, for 20 min at 37°C before being extensively washed. E-GFP negative and positive GEN2.2 were sorted on a MoFlow cytometer (Dako, Glostrup, Denmark). For the mixed leukocyte reaction, CpG matured allogeneic pDC were extensively washed and cultured in 96-well U-bottom plates at different cell numbers with 1 × 10^5 ^CFSE labelled CD45RA+^+ ^T-cells. On day 4, cells were harvested, washed, labelled for T specificity with anti-CD3 antibody and analysed by flow cytometry. The percentage of dividing T-cells was linearly correlated with the decrease in CFSE fluorescence.

### Activation of a MART-1 CD8^+ ^T cell clone by transduced DC subpopulations

Matured HLA-A2^+ ^DC subpopulations were obtained after transduction of cells by LV coding for a MART-1 peptide using a PGK promoter. Non-transduced matured HLA-A2^+ ^GEN2.2, CD34-pDC and Mo-DC and transduced GEN2.2 cells, CD34-pDC and Mo-DC were co-cultured in 96-well U-bottom plates at different ratios with 1 × 10^5 ^cells/well of a specific MART-1 CD8^+ ^T-cell clone HLA-A2 restricted (LT12) and labelled with CFSE as described earlier for the MLR. On day 5, cells were harvested, washed, labelled with an anti-CD8 antibody and analysed by flow cytometry. The percentage of dividing T-cells was linearly correlated with the loss in CFSE fluorescence.

### Flow cytometric analysis

The pDC phenotype was assessed using three color immunostaining with biotinylated, phycoerythrin (PE)-, Cy-Chrome (CyC)-and allophycocyanin (APC) -conjugated monoclonal anti-CD40 (5C3), anti-CD80 (L307.4), CD83 (HB15e), anti-CD86 (FUN-1), anti-HLA-DR (G46.6) antibodies (purchased from Becton Dickinson, Mountain View, CA, Pharmingen product, San Diego, CA) and anti-BDCA2 (AC-144), anti-BDCA4 (AD5-17F6) and anti-CD123 (AC145) (from Miltenyi Biotech). Data were acquired using a FACSCalibur flow cytometer (Becton Dickinson) and data analysis was performed using the CellQuest program (Becton Dickinson).

### Statistical analyses

Results were presented as the mean +/- standard deviation. Student's t-test for paired data was use to determine significant differences between the two groups. A p-value<0.05 was considered statistically significant.

## Results

### Transduction of pDC by LV and AAV vectors

**Figure 1:**
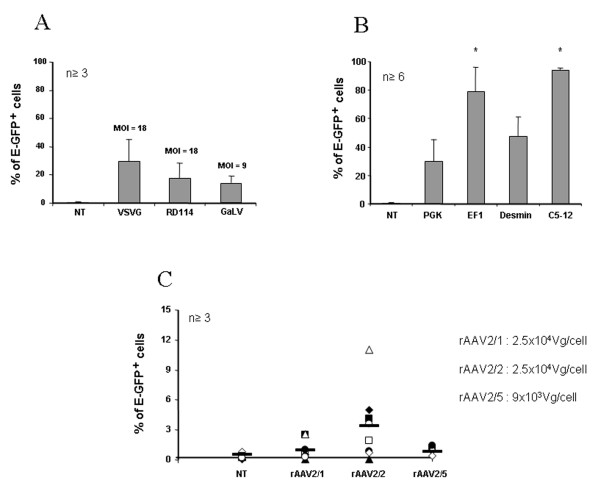
**Transduction efficiencies of GEN2.2**. The pDC cell line, GEN2.2, was non-transduced (NT) or transduced with E-GFP encoding vectors then analysed 5 days posttransduction. GEN2.2 were gated in forward/side scatter, then analyzed for the expression E-GFP by flow cytometry. (A) GEN2.2 were transduced by LV with a PGK promoter pseudotyped with either VSVG and RD114 envelopes at a MOI of 18 or with the GaLV envelope at a MOI of 9. (B) GEN2.2 were transduced with VSVG pseudotyped-LV with a PGK, EF1, desmin or C5–12 promoter, at a MOI of 18. (C) GEN2.2 were transduced by rAAV of serotype 1, 2 or 5 with a CMV promoter, with the number of viral genomes/cell indicated. Results are expressed as mean percentage of cell +/- SD over the number of independent experiments indicated.

**Figure 2:**
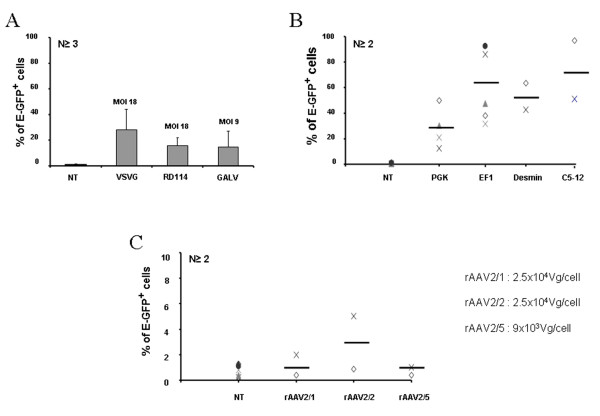
**Transduction efficiencies of CD34-pDC**. CD34-pDC were non-transduced (NT) or transduced with E-GFP encoding vectors at day 6 after the induction of differentiation, then cultured for 6 additional days. pDC were gated in forward/side scatter, then analyzed for the expression of E-GFP by flow cytometry. (A) CD34^+ ^progenitors were transduced by LV with a PGK promoter pseudotyped with either VSVG and RD114 envelopes at a MOI of 18 or with the GaLV envelope at a MOI of 9. (B) CD34^+ ^progenitors were transduced with VSVG pseudotyped-LV with a PGK, EF1, desmin or C5–12 promoter, at a MOI of 18. (C) CD34^+ ^progenitors were transduced by rAAV of serotype 1, 2 or 5 with a CMV promoter, with the number of viral genomes/cell indicated. Results are expressed as mean percentage of cell +/- SD over the number of independent experiments indicated.

We first compared the gene transfer efficiency into the human pDC cell line, GEN2.2, and in day 6 CD34-pDC, using LV pseudotyped with different envelopes from VSVG, GaLV or RD114 viruses. E-GFP expression can be easily and accurately monitored by FACS analysis. Preliminary experiments performed with LV encoding E-GFP under the control of the ubiquitous PGK promoter with different MOI (5–50), at a fixed cell density, showed that maximum transduction levels were reached at a MOI of 18 for VSVG-LV and RD114-LV and at a MOI of 9 for GaLV-LV (data not shown), without cellular toxicity. pDC were monitored for CD123, HLA-DR and E-GFP expression at day 5 to 6 posttransduction. A single exposure of GEN2.2 to VSVG-LV or RD114-LV led to 30% +/- 11.6% and 18.6% +/- 8% of cells which were E-GFP positive, respectively (figure 1A). When GaLV-LV was used, however, it was difficult in our hands to obtain high enough titers to reach a MOI of 18 using similar transduction conditions without cellular toxicity. So, at a two-fold lower MOI, a single exposure of GEN2.2 to GaLV-LV led to only 13.3% +/- 5.5% of E-GFP positive cells (figure 1A). Similar results were obtained on human CD34-pDC transduced at day 6 (figure 2A) and monitored 6 days posttransduction. Long-term expression of the transgene for GEN2.2 was maintained in all cases until at least day 60, as checked by flow cytometry (data not shown).

In a second step, we then selected the VSVG-LV pseudotype at MOI of 18 to transduce the pDC cell line, and evaluated the expression of GFP under the control of different promoters such as the ubiquitous PGK promoter, the hematopoietic cell-specific EF1 promoter, the muscle-specific desmin and the synthetic C512 promoters. The percentage of E-GFP^+ ^cells obtained was very high with both EF1 (79% +/- 15.3%) and C5–12 (94% +/- 2.8%) promoters which are 2.6 to 3 more efficient than the PGK promoter for transducing GEN2.2 (figure 1B). Surprisingly, a second muscle-specific promoter, desmin, was also highly efficient in pDC, since 47.7% +/- 11.1% of cells were E-GFP^+ ^(figure 1B). Similar results were obtained on human CD34-pDC transduced at day 6 (figure 2B) and monitored 6 days posttransduction. Altogether, these results show that VSVG-pseudotyped LV encoding the E-GFP as transgene under the control of the EF1 or C5–12 promoters are very efficient for transduction of pDC.

In a similar protocol, we used AAV vectors of different serotypes (rAAV2/1, rAAV2/2 and rAAV2/5) to transduce the pDC cell line and CD34-pDC and compared their efficacy. We previously showed that single-stranded rAAV2/1 and rAAV2/2 were very poorly efficient in transducing human pDC generated *in vitro *from CD34^+ ^progenitor cells [38]. We evaluated here, whether cells fully differentiated into pDC could be transduced by rAAV of serotypes 1 and 2, but also of serotype 5. Preliminary experiments performed with different amounts of viral particles (5 × 10^3 ^to 5 × 10^4 ^vg/cell), at a fixed cell density, showed that maximum transduction levels were reached with 2.5 × 10^4 ^vg/cell for rAAV2/1 and rAAV2/2 and with 9 × 10^3 ^vg/cell for rAAV2/5, with no cellular toxicity (data not shown). GEN2.2 and CD34-pDC were monitored for CD123, HLA-DR and E-GFP expression, but only at day 5 to 6 posttransduction, since pDC are dividing cells and AAV vectors are mainly episomal. A single exposure of pDC to rAAV2/1, rAAV2/2 or rAAV2/5 led to very low levels of transduced cells ranging from around 3% to less than 1% of E-GFP^+ ^cells (figure 1C and 2C). These results indicate that pDC are not susceptible to transduction by single-strand AAV vectors of serotype 1, 2 or 5.

### Immunophenotypical analysis of transduced pDC

**Figure 3:**
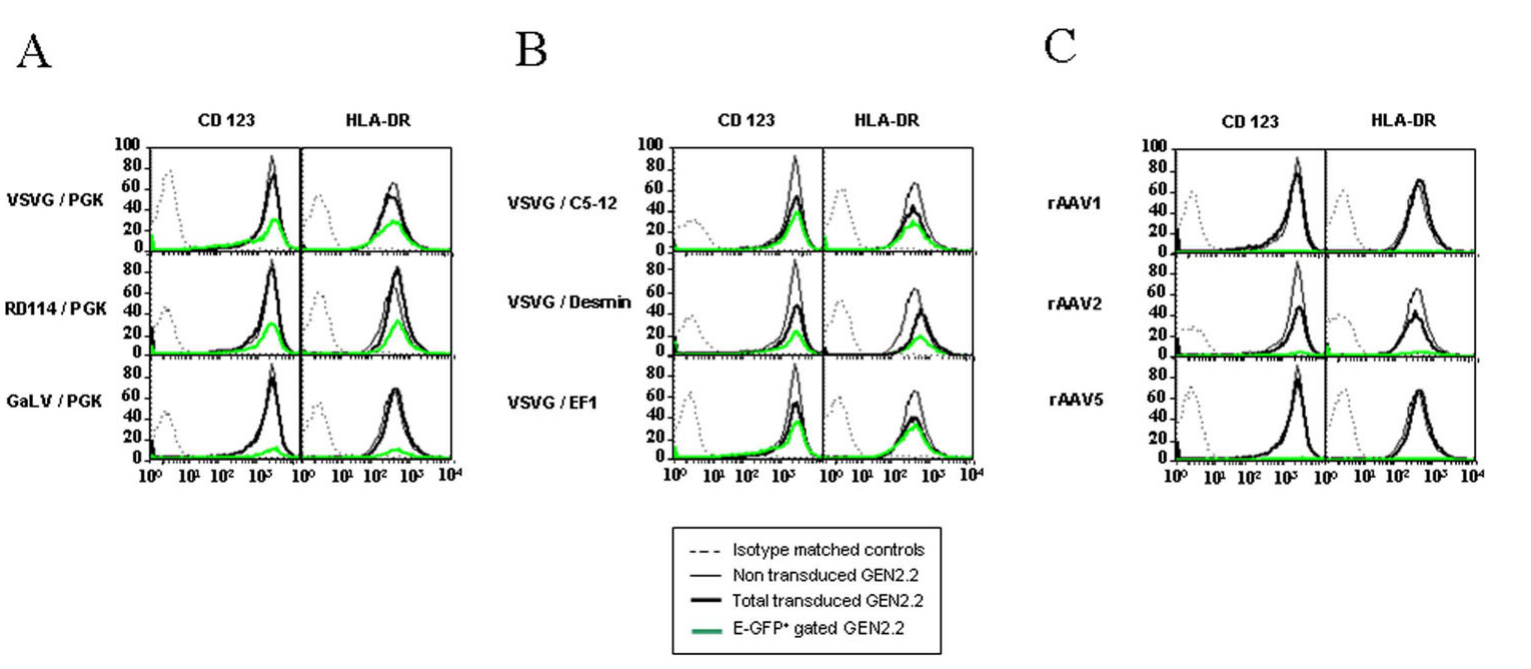
**Immunophenotype of transduced pDC**. Comparative phenotypes of transduced and untransduced GEN2.2 in absence of maturation agent, at day 5. Overlay histograms show the expression of CD123 or HLA-DR for untransduced (thin line), total transduced (thick line) and E-GFP^+ ^gated (green line) GEN2.2, versus isotype-matched controls (dotted line). (A) GEN2.2 transduced by LV with a PGK promoter pseudotyped with either VSVG, RD114 or GaLV envelopes. (B) GEN2.2 were transduced with VSVG pseudotyped-LV with a PGK, EF1, desmin or C5–12 promoter (C) GEN2.2 were transduced by rAAV of serotype 1, 2 or 5 with a CMV promoter. The results are representative of at least 4 experiments.

**Figure 4:**
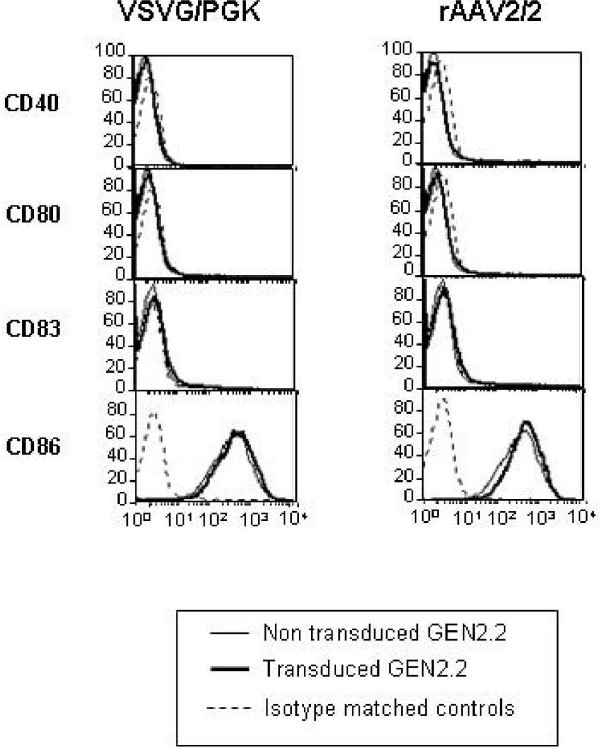
**Transduction of GEN2.2 does not induce maturation**. Comparative phenotype of transduced and non transduced GEN2.2. Overlay histograms show the expression of relevant antigens for untransduced (thin line) and transduced (thick line) with LV-VSVG/PGK or rAAV2/2, versus isotype-matched controls (dotted line). The results are representative of at least 3 experiments.

**Figure 5:**
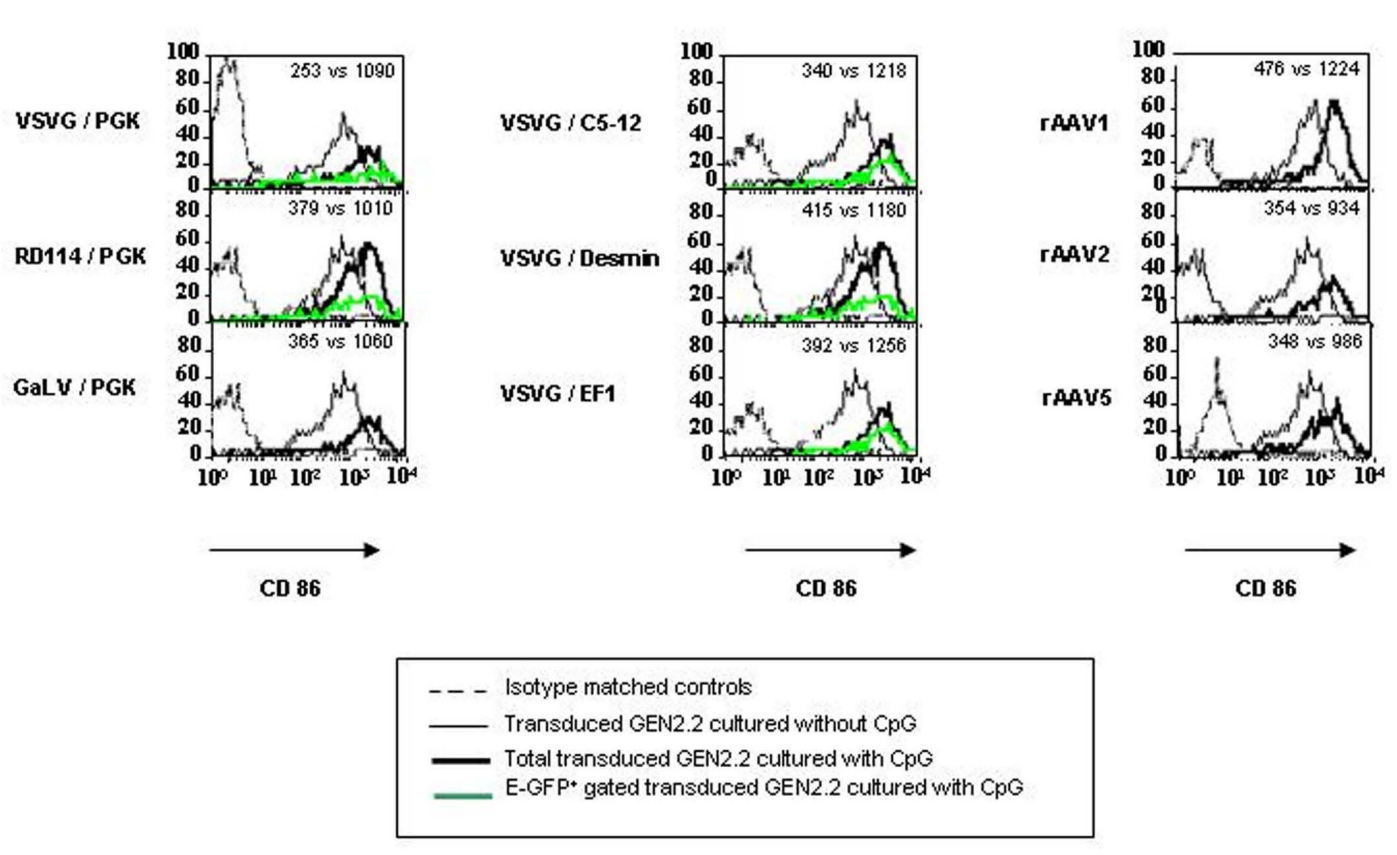
**CpG induced maturation of transduced pDC**. Comparative phenotype of transduced GEN2.2, 6 days posttransduction, in the absence and presence of CpG for 24 hours. Overlay histograms show the expression of relevant antigens for transduced GEN2.2 cultured without CpG (thin line), with CpG (thick line) and with CpG and gated on E-GFP^+ ^(green line) versus isotype-matched controls (dotted line). (A, B, C) Transduced GEN2.2 vectors are the same ones as those described in figure 2. Values indicated are MFI of the transduced populations cultured without CpG versus transduced populations cultured with CpG. The results are representative of at least 4 experiments.

The GEN2.2 cell line was previously characterized by its phenotype as a pDC cell line. These cells have been shown to express the human leukocyte antigen-DR (HLA-DR), the IL3-receptor (CD123) and the CD4 [18]. Moreover, as a hallmark of pDC, these cells are BDCA2 and BDCA4 (type II C lectin)-positive and CD11c- and CD1a-negative. Trypan blue exclusion and cell counting of LV and rAAV transduced GEN2.2 at the end of the culture period indicated that transduction had no deleterious effect on cell viability compared to control cells (data not shown). We explored in detail the immunophenotype of these transduced and control GEN2.2 by flow cytometry. We showed that whatever lentiviral or rAAV vectors used to transduced the GEN2.2 cells, no significant modification of the CD123 and HLA-DR expression (figure 3) of the CD4, BDCA2 and BDCA4 (data not shown) or of the costimulatory molecules and maturation marker CD80, CD86, CD40 and CD83 as illustrated figure 4, with two vectors, was observed, compared to control cells. Comparative phenotypic analysis of unactivated and CpG-activated transduced GEN2.2 revealed a normal upregulation of the co-stimulatory molecule CD86, demonstrating that the maturation capacity of transduced subpopulations was unaltered (Figure 5). Similarly, the phenotype of human transduced CD34-pDC was not modified compare to non-transduced cells (data not shown). Our results indicate that the LV transduction does not alter the phenotype of pDC or their capacity to mature.

### Functional properties of transduced pDC

**Figure 6:**
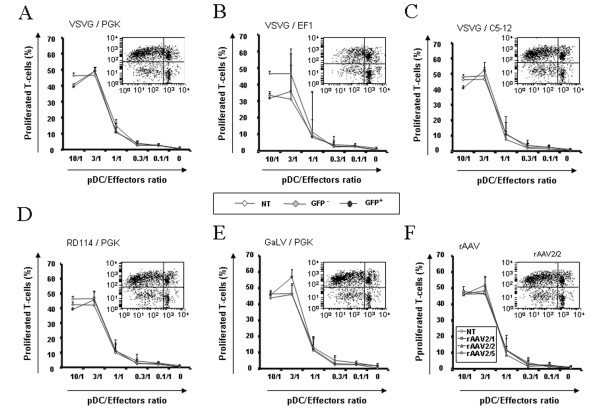
**T-cell stimulatory capacity of non-transduced and transduced GEN2.2 in mixed lymphocyte alloreactions**. Day 5 transduced GEN2.2 were matured in CpG for 24 hours, before cell sorting on an E-GFP expression basis. (A-E) Total non-transduced (NT), E-GFP^- ^and E-GFP^+^cell sorted GEN2.2 transduced by the same LV as those described in figure 3 were incubated with allogeneic T cells stained with CFSE. (F) Total non-transduced (NT) and rAAV2/1, rAAV2/2 or rAAV2/5 transduced unsorted GEN2.2 were incubated with allogeneic T-cells stained with CFSE. After 4 days of co-culture, percentages of CD3^+ ^dividing T cells measured by flow cytometry were linearly correlated with the loss of CFSE fluorescence. Dot plots inserted in graphs show one representative CFSE profile at the ratio 3/1 for GFP^+ ^cells. The data are shown as the means of 3 independent experiments.

**Figure 7:**
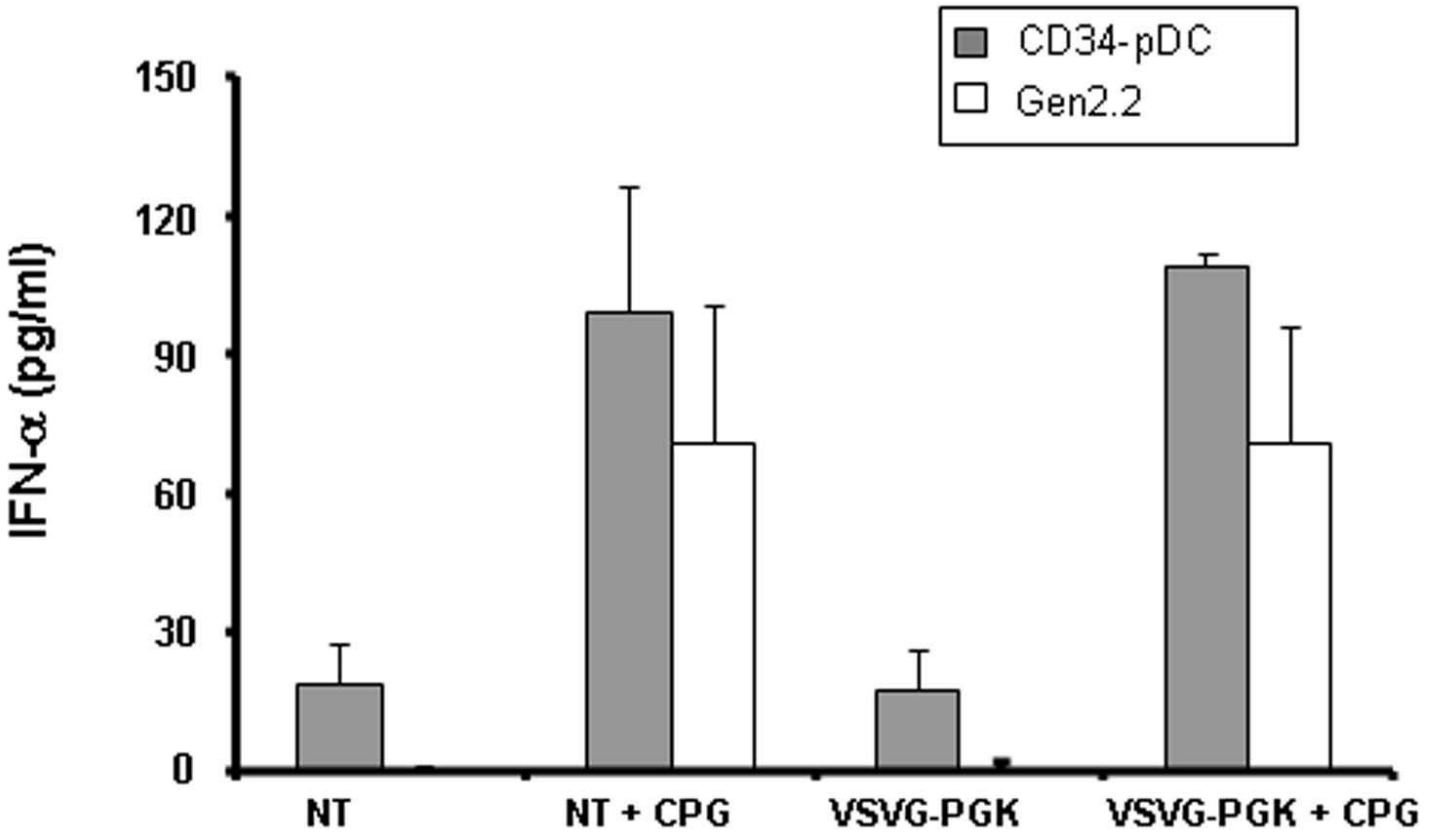
**IFN-α production by pDC**. GEN2.2 and day 6 CD34-pDC were non transduced (NT) or transduced by LV-VSVG at an MOI of 18 (LV-VSVG), then 6 days later, the IFNα production was measured in cell culture supernatants before or after maturation in CpG, for 24 hours. The data are shown as the means of 3 independent experiments.

**Figure 8:**
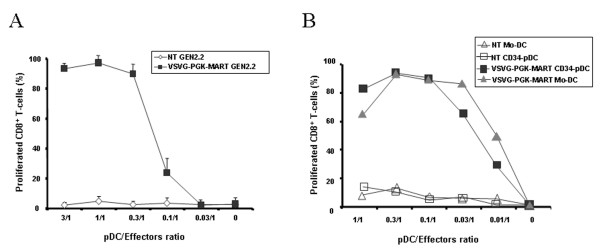
**CD8^+ ^T cell clone activation by LV transduced pDC**. *In vitro *antigen presentation capacities of LV transduced HLA-A2 pDC cells and Mo-DC. Cells were transduced with LV encoding the MART-1 peptide under the control of the PGK promoter. (A) Mature non-transduced (NT) and transduced (VSVG-PGK-MART-1) GEN2.2 or (B) CD34-pDC and Mo-DC were co-cultured with the HLA-A2 restricted CD8^+ ^T-cell clone specific for the MART-1 peptide (LT12) stained with CFSE. After 5 days of co-culture, percentages of CD8^+ ^dividing T-cells measured by flow cytometry were linearly correlated with the loss of CFSE fluorescence. The data in panel A are shown as the mean of triplicate and represent one out of 3 independent experiments whereas the data in panel B were performed once.

We evaluated the ability of different transduced GEN2.2 to stimulate allogeneic T cells in an allogeneic mixed lymphocyte reaction (MLR). GEN2.2 transduced with the different E-GFP encoding vectors were matured with CpG for 24 hours, then sorted by flow cytometry on the basis of E-GFP expression. Non-transduced, E-GFP negative and positive sorted GEN2.2 were used for stimulation of allogeneic T-cells labelled with CFSE. Both negative and positive E-GFP GEN2.2 populations displayed similar allostimulatory capacity compared to non-transduced GEN2.2, whatever vector used (figure 6 and data not shown). In response to these viruses, pDC are known to secrete high levels of type I IFN [14,15]. Of note, IFN-α was not detected in cell supernatant of any transduced GEN2.2 cultures when checked between 24 hours and 10 days following contact with the different viral particles. Nevertheless, GEN2.2 and CD34-pDC were always able to secrete IFN-α upon stimulation by the CpG motif via the toll-like receptor signalling pathway, as illustrated figure 7 for pDC transduced with a LV pseudotyped with VSVG coding for E-GFP under the control of the PGK promoter. Moreover, we evaluated the capacity of the HLA-A0201 expressing pDC to activate a CD8^+ ^T cell clone after transduction with a LV coding for the MART-1 peptide under the control of the PGK promoter. The transduced GEN2.2 obtained were efficient in activating a specific CD8^+ ^T-cell clone (Figure 8A). Results were confirmed on CD34-pDC transduced with the same LV expressing a MART-1 peptide (Figure 8B). Interestingly these transduced CD34-pDC were as efficient as Mo-DC for activation of a specific CD8^+ ^T cell clone (Figure 8B).

Altogether, these results indicate that the functional properties of pDC were not altered by LV or rAAV transduction. Furthermore, LV-transduced pDC were able to activate a CD8^+ ^T-cell clone.

## Discussion

The attractiveness of dendritic cells as a target for genetic manipulation is a consequence of their ability to initiate and orchestrate primary immune responses, including tolerogenic responses [1,45,46]. At least two circulating subsets of DC have been described: myeloid DC and pDC with evidence of functional differences in their ability to regulate the T-cell responses, to produce antiviral type I IFN and to cross-present exogenous antigens to CD8^+ ^T cells [47]. We previously showed that VSVG-pseudotyped HIV-1 vectors are good candidates for efficient transduction of monocyte- and CD34^+^-derived LC, without inducing phenotypic and functional maturation [26]. More recently, we also showed that self-complementary duplex strands but not single strands rAAV2/1 and 2 were also very efficient in transducing major DC subsets generated *in vitro*, including CD34^+^-derived pDC [38].

In this study, we extended LV transduction to pDC, using different pseudotyped HIV-1 vectors encoding E-GFP under the control of different promoters and showed that VSVG-pseudotyped LV encoding E-GFP under the control of EF1 or C512 promoters are the most efficient combinations, leading to transduction of 60% to 90% of the pDC cell line, GEN2.2 [18] and CD34-pDC. Of note, we showed that transduction did not alter alloreactive presentation properties of pDC. Furthermore, pDC transduced with LV expressing a MART-1 peptide was as efficient as Mo-DC for activation of a specific CD8^+ ^T cell clone. Altogether, these results show that antigen-loading of pDC through *ex-vivo *LV transduction may represent a relevant immunotherapy approach for particular clinical applications. Indeed, compared with antigen loading protocols using whole tumor cell lysates or recombinant tumor-associated antigen peptides, LV transduction offers the advantage of direct antigen processing from cytosolic proteins and of long lasting antigen expression.

Previous publications [30-32] reported efficient transduction levels of hematopoietic cells with LV pseudotyped with GaLV or RD114 envelopes. Here, the highest pDC transduction levels were obtained with the VSVG envelope, which was also previously shown to efficiently transduce human hematopoietic progenitor and leukaemia cells [26,48,49] as well as fully differentiated human monocyte-derived DC [50,51], with a long lasting expression. The EF1α promoter was shown to have a stronger activity than the PGK promoter in a human CD34^+ ^cell line [33] and in cultured cord blood cells [33,34] and allowed to obtain transgene-expressing myeloid DC [23]. Here, we showed that after a single exposure to VSVG-pseudotyped LV, the percentage of E-GFP expressing pDC was 2.6 fold higher when the expression was driven by the EF1 compared to the PGK promoter. The average copy number of the vector in transduced pDC under both conditions was similar (3–4 copies per cell), as determined by real-time quantitative PCR (data not shown). This indicates that the integration levels are similar with both constructions but that, as previously described, the promoter activity is different. We also evaluated two other promoters described to be muscle restricted [39-41], the desmin and synthetic C512 promoters which have been shown in gene therapy studies to specifically target muscles and to drive gene expression in a context of ss rAAV vectors [41]. As in our previous report [38], we showed here that even with an ubiquitous promoter like CMV, only a very low transduction efficiency could be reached with ss rAAV in the different DC subsets. So, in order to investigate the potential leak of these promoters in human DC subsets, we constructed and produced LV vectors carrying the two different cassettes. Surprisingly, we showed that the percentages of E-GFP expressing pDC with desmin and C512 promoters were very high and equivalent to those obtained with PGK and EF1 promoters, respectively. The average copy number in pDC for desmin and C512 promoters were 4 and 1 copies per cell, respectively, showing that the C512 promoters was at least as efficient as an ubiquitous promoter (data not shown). In contrast to the desmin promoter, the C512 promoter was also active in monocyte-derived DC and LC (around 10% of E-GFP^+ ^cells) and in a human colorectal carcinoma (HCT116) (data not shown). Nevertheless, transgene expression with these cassettes in ss AAV vectors was not detectable (data not shown). Taken together, these data suggest that the use of desmin or C5–12 promoters in ss rAAV, for clinical gene therapy protocols, will not induce transgene expression in DC subsets. Nevertheless, the use of these promoters in sc rAAV, which are highly efficient for transducing major DC subsets [38] might elicit high immune responses against the transgene.

## Conclusion

DC transduction with LV preparations can serve as vaccine vehicles in human through efficient transduction levels and are also useful *in vitro *to evaluate the immunogenicity of the vector preparations and the specificity and safety of promoters used in gene therapy protocols.

## Competing interests

The authors declare that they have no competing interests.

## Authors' contributions

VP contributed to the experimental design, data acquisition and analysis, and drafting of the manuscript. BS contributed to the data acquisition and analysis. MS designed lentiviral vector constructions. CL provided the Gen2.2 cell line. PJ provided the Gen2.2 cell line and critically revised the manuscript. DJ gave the final approval of the version to be published. MC conceived of the study, participated in its design and coordination and drafted the manuscript. All authors read and approved the final manuscript.
